# Associations between adherence to MIND diet and severity, duration and frequency of migraine headaches among migraine patients

**DOI:** 10.1186/s13104-020-05181-4

**Published:** 2020-07-16

**Authors:** Moein Askarpour, Habib yarizadeh, Ali Sheikhi, Faezeh Khorsha, Khadijeh Mirzaei

**Affiliations:** 1 Department of Community Nutrition, School of Nutritional Sciences and Dietetics, Tehran University of Medical Sciences (TUMS), P.O. Box: 14155-6117, Tehran, Iran; 2 Students’ Scientific Research Center (SSRC), Tehran University of Medical Sciences (TUMS), Tehran, Iran

**Keywords:** Migraine headache, Mind diet, Visual analog scale, Migraine Disability Assessment Questionnaire

## Abstract

**Objectives:**

Migraine is a neurological disorder causing unbearable pain. Dietary approach is proposed as a preventive way of reducing the severity of migraine headaches. The present study aimed to examine the association between MIND diet and migraine headaches.

**Results:**

We found that participants with higher score of MIND diet compared to those with lower score, were less likely to have severe headaches (OR = 0.64; 95% CI 0.45, 0.91; *P *= 0.01). Moreover, our results showed an inversed correlation between mind diet score and duration (β = − 0.14, 95% CI − 1.42, − 0.14, *P *= 0.04) and frequency of headaches (β = − 0.13, 95% CI − 0.99, − 0.07, *P *= 0.03).

## Introduction

Migraine is a common neurovascular brain disorder that often causes throbbing headaches of varying intensity, lasting hours to days [1]. It is often accompanied by complications such as vomiting and photophobia [2]. Migraine is estimated to affect 10% of the global population, more prevalent in women than men [3]. The disability caused by migraine is substantial and could impose a great economic burden upon societies [4]. Moreover, Migraineurs are at increased risk of cardiovascular disorders and mortality [5]. Therefore, finding the best approach to control and manage this disease is of utmost importance. Though the exact mechanism behind this disease is not fully clear, changes in the blood flow of the brain are considered to be the main stimuli. Several medications have been introduced to alleviate the symptoms [6], but given the major side effects that these medications may create [7], identifying disease-modifying risk factors to prevent headaches is crucially important. Research has shown that nutrition may have a role.

With the population of elderly growing, the rate of age-related diseases like dementia is expected to rise, making it a high-priority problem. Though there is no effective treatment for dementia, recent research has shown that the Mediterranean and Dietary Approaches to Stop Hypertension (DASH) diets could be efficacious in curtailing cognitive decline. Recently, a hybrid of these two dietary patterns, named the MIND diet, has been developed by Morris et al. [8]. The MIND diet focuses on natural plant-based foods and limits the intake of animal and high saturated fat foods. It also emphasizes the consumption of berries and green leafy vegetables which are known to have antioxidant and anti-inflammatory properties and inhibit neurotoxic death. The beneficial effect of the MIND diet on Alzheimer’s dementia [8], depressive symptoms [9], and parkinsonian signs [10] have shown in previous studies. Though studies examining the effect of MIND diet on cognitive function are a handful, they have shown that adherence to the MIND diet was associated with slower rates of cognitive decline [11]. Since previous studies have linked migraine to inflammation and oxidative stress [12–14], we propose this hypothesis that the MIND diet could have a potential beneficial effect on migraine.

To our best knowledge, no study has ever investigated the relationship between the MIND diet and migraine, we decided to conduct this cross-sectional observational study to examine the effect of MIND diet on intensity, duration and frequency of migraine headaches.

## Main text

### Methods

#### Participants

In this cross-sectional study, 266 women aged 18–50 with history of migraine headaches were selected from two neurology clinics in Tehran. The sample size was computed according to the following formula:

$$ {\text{n}} = \left( {\left( {\left[ {\left( {{\text{Z}} _{{1 -\upalpha}} + {\text{Z}} _{{1 -\upbeta}} } \right) \times \surd 1 - {\text{r}}^{2} } \right]/{\text{r}}} \right)^{2} + 2} \right), $$

which r = 0.25, β = 0.95, and α = 0.05 [15]. Based on this formula, 241 individuals were required; however, due to the availability of data and to account for any possible exclusion, 266 individuals were included.

In the present study, Inclusion criteria were the following: participants with history of migraine headaches diagnosed by an expert neurologist, voluntarily involvement, BMI ranged between 18.5 and 30, and visiting the clinics for the first time. Consent forms were collected by all of the participants.

#### MIND diet assessment

Dietary intake was evaluated by a face-to-face interview using a 147-items semi-quantitative food frequency questionnaire (FFQ), which its reliability and validity had been already approved in Iran [16]. To compute the MIND diet scores, some dietary components of the FFQ was used in this study which are presented in Table 1. Of 15 dietary factors in the original scoring of MIND diet, 10 of them were defined as brain healthy food groups (green leafy vegetables, other vegetables, nuts, berries, beans, whole grains, fish, poultry, olive oil, and wine) and 5 as brain unhealthy food groups (red meats, butter and stick margarine, cheese, pastries and sweets, and fried/fast food) [11]. However, in the current study we used a modified MIND diet scoring based on Iranian food habits [9]. In this scoring, wine consumption was not considered (forbidden drinking). The other 14 food groups were applied in the MIND scoring. First, individuals were categorized according to tertile categories of dietary intakes. Participants in the bottom tertile of brain healthy food groups were given the score of 0, those in the middle tertile were given the score of 0.5 and individuals in the top tertile were given the score of 1. On the other hand, we did vice versa about brain unhealthy food groups. Finally, the overall score was computed by summing up of all dietary components scores. Therefore, the participant’s MIND diet score ranged from 0 to 14.

**Table 1:** Components of MIND diet

Brain healthy foods	
Green leafy vegetables	Greens, lettuce
Other vegetables	Cabbage, raw carrot, potato, peas or lima beans, green/red peppers, eggplant, onion, cucumber, tomatoes, tomato sauce
Berries	Strawberries (strawberry, fresh berries)
Nuts	Walnuts, almonds, peanuts, pistachios, hazelnuts
Whole grains	Dark bread (Iranian)
Fish (not fried)	Fish
Beans	Beans, mung bean, peas, lentils, chickpea
Poultry (not fried)	Chicken

Brain unhealthy foods	
Butter, margarine	Butter, margarine, animal fats
Cheese	Cheese
Red meat and products	Red meat, hamburger, sausages
Fast fried foods	French fries, pizza
Pastries and sweets	Biscuit, ice cream, cake, confections, cocoa, Gaz (an Iranian confectionery made of sugar, nuts and tamarisk), chocolate

#### MIDAS and VAS questionnaires

To assess headache-related disability, the Migraine Disability Assessment (MIDAS) questionnaire was applied. The MIDAS questionnaire validity and reliability already were assessed in Iranian population [17]. The VAS score cut-off points ranged from 1 to 10 and categorized pain severity in three levels: 1 to 3 (mild pain), 4 to 7 (moderate pain) and 8 to 10 (severe pain) [18].

#### Assessment of other variables

Weight was measured via a digital scale (SECA, Hamburg, Germany) to the nearest 0.1 kg, while wearing no shoes and one layer of clothing. To examine physical activity (PA), International Physical Activity Questionnaire (IPAC), was used [19]. PA was shown as metabolic equivalent hours per week (METs h/week).

#### Statistical analyses

The Chi-square test was used to assess the association between the quartiles of MIND diet and qualitative variables. In addition, one-way analysis of variance (ANOVA) was used to assess the association between the quartiles of MIND diet and quantitative variables. Covariance analysis (ANCOVA) was applied to compare the dietary intakes of participants among quartiles of MIND diet by adjusting the effect of total energy. Confounding factors including total energy, age, BMI, PA, smoking status, water intake, salt intake, medication usage, and menstruation headaches were applied in adjusted models. To assess the association between the MIND score quartiles and the headache severity and disability, multinomial logistic regression model was used in crude and adjusted models. Moreover, to determine the association between headache duration and frequency (dependent variable) and the quartiles of MIND diet (independent variables), Linear Regression Models analysis was used, in crude and adjusted models. P < 0.05 was considered as level of statistically significance. SPSS version 24 (SPSS Inc., Chicago, IL, USA) was used to perform statistical analysis.

### Results

#### Study population characteristics

The baseline characteristics of the participants among quartiles of MIND diet are shown in Table 2. The mean (± SD) age, height, weight, BMI and physical activity of participants were 34.32 (7.86) years, 1.61 (0.05) m, 69.41 (13.02) kg, 26.50 (4.88) kg/m^2^, 407.73 (519.13) MET/min/week, respectively. Quantitative and qualitative variables across MIND diet quartiles did not show any significant differences. The percentages of study population among the MIND diet quartiles were: Q_1_ 27.4, Q_2_ 25.5, Q_3_ 25.5, and Q_4_ 21.6.

**Table 2:** General characteristics of study population among quartiles (Q) of MIND diet score

	Quartiles of the MIND diet score					
	Total (N = 266)	Q_1_ (n = 73) ^a^4.12	Q_2_ (n = 68) ^a^6.14	Q_3_ (n = 68) ^a^8.07	Q_4_ (n = 57) ^a^10.21	*P-value
Age (years)	34.32 ± 7.86	34.57 ± 8.43	34.21 ± 8.17	34.55 ± 6.69	33.71 ± 8.24	0.87
Height (m)	1.61 ± 0.05	1.62 ± 0.05	1.62 ± 0.05	1.61 ± 0.05	1.62 ± 0.04	0.48
Weight (kg)	69.41 ± 13.02	67.34 ± 13.47	70.27 ± 13.57	68.57 ± 12.12	72.27 ± 13.28	0.27
BMI (kg/m^2^)	26.50 ± 4.88	25.87 ± 4.73	26.97 ± 5.45	26.18 ± 4.86	27.38 ± 4.56	0.29
PA (MET-h/week)	407.73 ± 519.13	410.73 ± 724.27	449.41 ± 410.83	412.02 ± 420.24	452.25 ± 471.23	0.78
Current smoker (n (%))						0.11
Yes	13 (4.9)	5 (1.9)	0 (0)	3 (1.1)	5 (1.9)	
No	253 (95.1)	68 (25.5)	68 (25.5)	65 (24.1)	52 (20)	
Marital status (n (%))						0.75
Single	74 (27.8)	21 (7.9)	20 (7.5)	16 (6)	17 (6.4)	
Married	192 (72.2)	52 (19.5)	48 (18.1)	52 (19.5)	40 (15.1)	
Education status (n (%))						0.87
≤ Diploma	104 (39.1)	30 (12.3)	26 (9.7)	24 (9.1)	24 (9.1)	
> Diploma	162 (60.9)	43 (16.1)	42 (15.6)	44 (16.5)	33 (12.7)	
Medication use (n (%))						0.27
Yes	127 (47.7)	32 (12.1)	28 (10.5)	37 (13.9)	30 (11.2)	
No	139 (52.3)	41 (15.4)	40 (15)	31 (11.7)	27 (10.2)	
Family history of migraine (n (%))						0.73
Yes	173 (65)	51 (19.1)	45 (16.9)	45 (16.9)	33 (12.1)	
No	92 (35)	22 (8.4)	23 (8.7)	23 (8.7)	24 (9.2)	

Quantitative variables (age, height, weight, BMI, PA, headache duration) reported as Mean ± SD

Qualitative variables (current smoker, education and marital) reported as number (%)

*BMI* body mass index, *PA* physical activity

*Chi-square test and ANOVA were applied for qualitative and quantitative variables, respectively

^a^ Median MIND diet score

#### Dietary intake and MIND diet quartiles

Dietary intakes of the participants among MIND diet quartiles are presented in Additional file 1: Table S1. Participants in the highest quartile of MIND diet had higher consumption of vegetables, legumes and nut, while had lower intake of refined grain and energy intake compared to the lowest quartile of MIND diet score. However, the consumption of fruits, dairy, whole grain, meat, and water intake across quartiles of MIND diet did not show any significant differences.

#### MIND diet and migraine headache

The association between duration, frequency, severity and disability of migraine headache across quartiles of MIND diet in crude model and adjusted model are shown in Table 3. In the crude model of multinomial logistic regression, greatest adherence to the MIND diet did not show a significant relation with severity of disability based on MIDAS score (OR = 0.97; 95% CI 0.58, 1.52; *P *= 0.93). Even after adjustment for potential confounding factors including energy intake, age, BMI, PA, smoking status, water intake, salt intake, menstruation headaches, and medication usage, no association was observed (OR = 0.96; 95% CI 0.59, 1.49; *P *= 0.81). A significant inverse association was observed between MIND diet score and odds of severe headaches in crude model (OR = 0.69; 95% CI 0.47, 0.98; *P *= 0.04). After adjustment for the potential confounding factors, individuals in the highest quartile of MIND diet were 36% less likely to have severe headaches (OR = 0.64; 95% CI 0.45, 0.91; *P *= 0.01) compared with those in the lowest quartile.

**Table 3:** Crude and multivariable-adjusted odds ratios for severity, disability, duration and frequency of migraine headaches across MIND diet scores

	Crude models		*P*-value	Adjusted models		^b^*P*-value
	OR	(0.95% CI)		OR	(0.95% CI)	
VAS						
Mild pain^c^	–	–	–	–	–	–
Moderate pain	0.81	(0.61, 1.12)	0.21	0.85	(0.65, 1.14)	0.28
Sever pain	0.69	(0.47, 0.98)	0.04	0.64	(0.45, 0.91)	0.01
MIDAS						
Without disability^c^	–	–	–	–	–	–
Mild disability	0.91	(0.57, 1.24)	0.72	0.93	(0.61, 1.27)	0.77
Moderate disability	0.90	(0.57, 1.27)	0.68	0.85	(0.54, 1.19)	0.28
Severe disability	0.96	(0.59, 1.49)	0.81	0.97	(0.58, 1.52)	0.93
Headache duration of each attack	^a^− 0.16	(− 1.43, − 0.31)	0.01	^a^− 0.14	(− 1.42, − 0.14)	0.04
Frequency of headache in month	^a^− 0.14	(− 1.01, − 0.12)	0.02	^a^− 0.13	(− 0.99, − 0.07)	0.03

*MIDAS* Migraine Disability Assessment Questionnaire, *VAS* visual analog scale

^a^ The β coefficient has been shown

^b^ Adjusted for confounders

^c^ Considered as reference group

Linear Regression Models analysis (LRM) showed an inverse association between MIND diet score and duration of migraine headaches in crude (β = − 0.16, 95% CI − 1.43, − 0.31 *P *= 0.01), and adjusted models (β = − 0.14, 95% CI − 1.42, − 0.14, *P *= 0.04). Moreover, frequency of headaches indicated an indirect correlation with MIND diet score in crude model (β = − 0.14, 95% CI − 1.01, − 0.12, *P *= 0.02), and adjusted model (β = − 0.13, 95% CI − 0.99, − 0.07, *P *= 0.03).

### Discussion

To the best of our knowledge, the present study is the first to examine the association between the modified MIND diet and migraine headaches. The main analyses showed that higher adherence to the modified MIND diet is related to lower headache intensity, duration, and frequency among migraine patients. However, the MIDAS score, which assesses the disability caused by migraine, did not have a significant relation with the modified MIND diet.

There are some possible mechanisms through which the effect of the MIND diet on migraine could be explained. Mainstream medicine views migraine headaches as a result of the excitation of sensory fibers in the brain due to neurogenic inflammation which happens in response to even normal stimuli. The notion of migraine being a neuroinflammatory disease has been strengthened by studies investigating neuropeptides such as calcitonin gene-related peptide (CGRP). CGRP is known to be involved in several physiological processes including the dilation of cerebral and dural blood vessels and the release of inflammatory mediators [20]. Now that the current evidence points out that CGRP plays a central role in the pathophysiology of migraine, blocking the effects of it seems to be a logical clinical approach, as anti-CGRP drugs administered for migraine, such as Triptan, have proven fairly successful in attenuating headaches [21]. Dietary antioxidants could also impose similar effects if consumed in adequate amounts. One study demonstrated that rats orally fed with grape seed extract for 2 weeks had lower expression of CGRP than control rats [22]. Moreover, dietary components in vegetables such as Indole-3-carbinol and sulforaphane could inhibit the activation of CGRP receptor signaling [23]. Mediterranean and DASH diets have long been associated with lower levels of oxidative stress and inflammation [24–26]. The antioxidants in berries and the vitamin E in olive oil, green leafy vegetables and nuts could ease migraine by protecting the brain from oxidative stress [27]. Additionally, the omega-3 fatty acids found in fish may benefit migraine by lowering inflammation in the brain [28].

Low magnesium level has been attributed to causing cerebrovascular constriction and increased vascular reactivity and membrane receptor activity to mediators, producing headaches. Accordingly, several studies have shown that migraineurs usually have low brain magnesium levels during migraine attacks and may also suffer from a magnesium deficiency [29, 30]. It is worth noting that certain food items (such as green leafy vegetables, nuts, and seeds, legumes) which are integral parts of the MIND diet, are rich sources of magnesium.

Several studies are suggesting a positive association between hypertension and migraine [31–33]. Malignant hypertension, through imposing pressure on cranium, could be responsible for causing headaches. Research showing the effect of DASH and Mediterranean diets on lowering blood pressure is abundant [15].

## Conclusion

We found that higher adherence to the modified MIND diet may reduce the severity of pain in migraine patients. These initial findings need to be confirmed with prospective studies in order to confirm the relationship between the modified MIND diet and migraine.

## Limitation

The number of enlisted subjects were relatively low. The absence of men is another major limitation that is worth noting. The cross-sectional design of the study prohibits us from inferring causality. Finally, questionnaire responses are subjectively based on participant’s memory and their perception of pain.

## Supplementary information

**Additional file 1: Table S1.** Dietary intake of study population among quartiles (Q) of PRAL and NEAP.

## Data Availability

The data are not publicly available due to containing information that could compromise the privacy of research participants.

## Abbreviations

ANOVA: One-way analysis of variance
FFQ: Food frequency questionnaire
MIDAS: Migraine Disability Assessment
IPAC: International Physical Activity Questionnaire
PA: Physical activity
LRM: Linear Regression Models
TUMS: Tehran University of Medical Science
VAS: Visual analog scale
