# Supplementary material for: Associations between adherence to MIND diet and severity, duration and frequency of migraine headaches among migraine patients
Source: BMC Res Notes. 2020 Jul 16;13:341. doi: 10.1186/s13104-020-05181-4 (PMC7364542; doi:10.1186/s13104-020-05181-4)
Supplement: Supplementary file 1 — Additional file 1: Table S1. Dietary intake of study population among quartiles (Q) of PRAL and NEAP. [file 13104_2020_5181_MOESM1_ESM.docx]

**Table S1.** Dietary intake of study population among quartiles (Q) of PRAL and NEAP

| Quartiles of the MIND diet score | | | | | |
| --- | --- | --- | --- | --- | --- |
|  | **Q_1_**  (n=73) | **Q_2_**  (n=68) | **Q_3_**  (n=68) | **Q_4_**  (n=57) | ****P*-value** |
| Energy (kcal/d) | 2345.92±505.12 | 2310.36±431.12 | 2110.40±710.87 | 1947.24±452.89 | <0.001 |
| Vegetables (g/d) | 136.87±44.35 | 195.24±124.21 | 278.21±24.79 | 361.22±87.21 | <0.001 |
| Fruits (g/d) | 312.24±178.37 | 341.24±243.56 | 327.21±248.01 | 291.15±151.12 | 0.59 |
| Dairy (g/d) | 310.12±87.24 | 287.12±124.24 | 251.12±96.54 | 271.12±78.24 | 0.09 |
| Legumes and nut (g/d) | 36.12±25.24 | 41.12±26.12 | 68.12±47.45 | 84.12±26.56 | <0.001 |
| Refined grain (g/d) | 381.12±145.24 | 367.12±125.14 | 312.21±154.22 | 187.24±64.12 | <0.001 |
| Whole grain (g/d) | 49.21±56.45 | 68.24±57.24 | 43.21±57.54 | 56.24±34.21 | 0.09 |
| Meat (g/d) | 54.57±24.21 | 55.25±28.12 | 58.58±34.36 | 60.01±32.68 | 0.51 |
| Water intake (L/d) | 1.31±0.81 | 1.11±0.27 | 1.23±0.87 | 1.01±0.29 | 0.48 |

* Based on ANCOVA, adjusted for total energy except total energy and water intake.

Mean ± SD (all such values).
